# 2- and 3-dimensional synthetic large-scale *de novo* patterning by mammalian cells through phase separation

**DOI:** 10.1038/srep20664

**Published:** 2016-02-09

**Authors:** Elise Cachat, Weijia Liu, Kim C. Martin, Xiaofei Yuan, Huabing Yin, Peter Hohenstein, Jamie A. Davies

**Affiliations:** 1 Centre for Integrative Physiology & Synthsys Centre for Synthetic & Systems Biology, University of Edinburgh, George Square, Edinburgh EH8 9XB, UK; 2 School of Engineering, University of Glasgow, Rankine Building, Oakfield Avenue, Glasgow G12 8LT, UK; 3 The Roslin Institute, University of Edinburgh, Easter Bush Campus, Midlothian, EH25 9RG, UK

## Abstract

Synthetic biology provides an opportunity for the construction and exploration of alternative solutions to biological problems - solutions different from those chosen by natural life. To this end, synthetic biologists have built new sensory systems, cellular memories, and alternative genetic codes. There is a growing interest in applying synthetic approaches to multicellular systems, especially in relation to multicellular self-organization. Here we describe a synthetic biological system that confers large-scale *de novo* patterning activity on 2-D and 3-D populations of mammalian cells. Instead of using the reaction-diffusion mechanisms common in real embryos, our system uses cadherin-mediated phase separation, inspired by the known phenomenon of cadherin-based sorting. An engineered self-organizing, large-scale patterning system requiring no prior spatial cue may be a significant step towards the construction of self-assembling synthetic tissues.

Synthetic biology allows researchers to create and explore biological processes alternative to those that arose during the natural evolution of life on Earth. Examples include the creation of new sensory systems^1^, new metabolic pathways^2^, new information storage systems^3^ and alternative genetic codes^4^. These have been made in unicells but there is now intense interest in engineering alternative mechanisms for patterning and organization of multicellular systems^5^. We have therefore designed a *de novo* patterning system for mammalian cells based on phase separation, an alternative to the reaction-diffusion (‘Turing’/ ‘Meinhardt’) mechanisms thought to pattern fields in natural embryos^6^,^7^. An engineered patterning system that requires no prior cue (e.g. inducer gradient) is a significant step forward in the synthetic biology of multicellular systems.

The development of multicellular organisms rests on three processes: pattern formation, differentiation and morphogenesis. Of these, differentiation, which is driven by changes in transcription, has been the easiest to achieve in engineered systems, through synthetic control of gene expression^8^. Progress has recently been made towards synthetic morphogenesis with the production of a library of morphogenetic effector modules for use in mammalian cells^9^. Pattern formation has received less attention, and most engineers of synthetic patterning mechanisms, in bacteria or mammalian cells, have concentrated on elaboration of existing patterns (e.g. experimentally-applied gradients) rather than creation of patterns *de novo*^10^,^11^,^12^,^13^. These gradients are either applied directly, or emerge from colony edge effects^14^. In natural systems, true *de novo* patterning of cell fields is usually assumed to occur by the reaction-diffusion mechanism proposed by Turing^6^ (reviewed in the context of modern biology by Ball^15^) and by Gierer and Meinhardt^16^. Reaction-diffusion mechanisms use coupled positive and negative feedback: a slowly-diffusing ‘activator’ molecule encourages synthesis of more activator and also of a quickly-diffusing inhibitor. The result, for suitably chosen parameters of the system, is spatial patterning in which zones (spots, stripes) where there is more activator than inhibitor are separated by zones in which the opposite is true. Although the reaction-diffusion idea is promising, attempts to build it have not yet resulted in a working system^17^. This might be because the operation of such systems can be fairly parameter-sensitive. Also, at least in animal systems, cell movement may interfere with the establishment of pattern when movement is fast compared to the response times in the feedback systems.

An alternative approach to spontaneous patterning is the use of phase separation, a mechanism in which two (or more) constituents of a mixture have, or acquire at some moment, distinct properties that cause the mixture to separate. A well known example is a mixture of water and oil which, if left alone in a bulk-liquid form (e.g. in a test-tube), will separate into two layers to allow most of the water molecules form energetically favourable hydrogen bonds with one another (they cannot form them with oil). When movement is in some way constrained, for example in a thin film of liquid, complete separation may not be possible and phase separation produces patches of one phase, separated and prevented from coalescing by zones of the other phase: alternating patches of water and water-free zones on a waxed windscreen is an example familiar to motorists. Phase separation is receiving increasing attention in explaining patterns at an ecosystem scale^18^. Mussels, for example, can be in high- or low-motility phases: high population densities cause individuals to exhibit low motility, so that exploring individuals become ‘trapped’ where many of their kind already are, depleting intervening spaces^18^. This generates alternative patterns of mussel beds and spaces. Similar action has been reported in a bacterial system^19^.

Cells bearing different adhesion molecules, or different quantities of the same adhesion molecule, naturally undergo a phase separation^20^,^21^. Like interfaces between water and oil, the interfaces between the cells exhibit properties of surface tension^22^. Numerous papers have investigated cell sorting through differential adhesion *in vitro*^23^,^24^,^25^. Generally, this phase separation has been explored in systems that are small enough, and permissive enough of movement, for phases to separate completely. We wondered whether it would be possible to construct an inducible mechanism for patterning by phase separation in mammalian cells, which could operate on larger scales and generate spots or stripes. Compared with reaction-diffusion systems, phase separation systems have the advantage that pattern generating action is less dependent on parameters being in the correct range (although the parameters do still control the nature of the pattern formed and the speed with which it forms). They also have the feature that they depend on cell movement, rather than being destabilized by it. Phase-separation may therefore be of greatest use in animal-cell systems, whereas reaction diffusion may be more appropriate to plants.

## Results

### Construction of cells for phase separation

To implement the patterning system described above in real mammalian cells, we extended our published library of morphogenetic modules^9^ to include more than one cadherin. Cadherins are a family of over 100 calcium-dependent cell adhesion molecules, the ‘classical’ members of which can bind both homotypically (cadherins on one cell binding to identical cadherins on another cell) or heterotypically (cadherins of one type binding to cadherins of another type on another cell)^26^. Usually, cell-cell adhesion using homotypic cadherin interactions is stronger than that using heterotypic interactions^27^.

**Figure 1:**
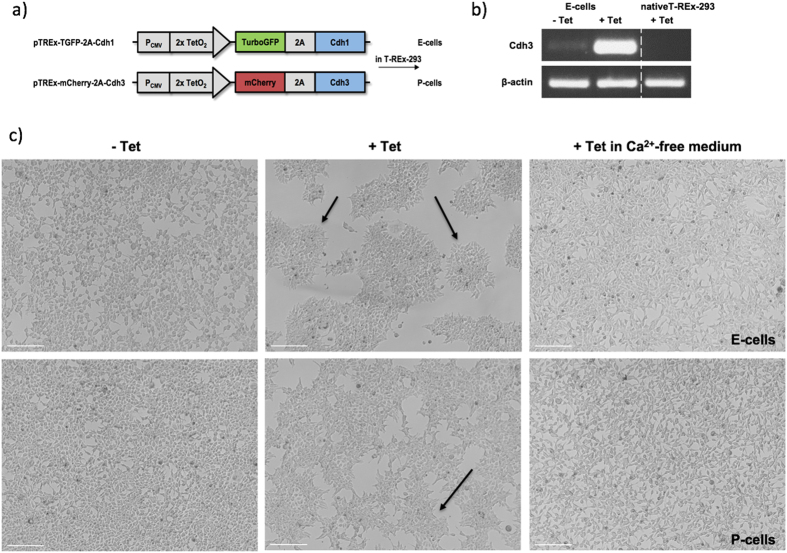
Introduction to the tools used to construct a phase-separation-based patterning system in living cells. (**a**) Cdh1 and Cdh3 constructs were used to transfect T-REx-293 cells to produce E-cells (characterized in a previous publication:^9^) and P-cells. (**b**) Levels of murine Cdh3 transcripts in P-cells induced (‘ + Tet’) or uninduced (‘−Tet’) with tetracycline for 48 h and in T-Rex-293 wild-type cells in the presence of tetracycline, confirming inducibility. The band showing β-actin, a ‘housekeeping’ protein that should be unaffected by tetracycline, is a control to demonstrate equal loading of the RT-PCR reaction and gel. A similar analysis for the Cdh1 transcript has been presented in^9^. (**c**) Comparison of E-cell and P-cell cell morphology after 48 h of culture with or without tetracycline. In the absence of tetracycline, cells were scattered with no obvious adhesive islands. Following induction, adhesive islands formed (arrows), being more smooth-edged in E-cells than in P-cells. The adhesion clusters observed in the presence of tetracycline were disrupted in calcium-free medium, as would be expected for a cadherin-dependent mechanism. Scale bars: 200 μm.

The morphogenetic module library^9^ already contained a tetracycline-inducible Cdh1 (E-cadherin)-mediated adhesion module, *pTREx-GFP-2A-Cdh1.* We added a similar Cdh3 (P-cadherin) module, *pTREx-mCherry-2A-Cdh3* to the library (Fig. 1a) because the differential adhesive strengths between homotypic and heterotypic E-cadherin and P-cadherin interactions have been reported to drive cell sorting when these cadherins are expressed in mammalian cells^27^. The *pTREx-mCherry-2A-Cdh3* construct was transfected in human T-Rex-293 cells, as was done previously with *pTREx-GFP-2A-Cdh1*^9^. Expression was confirmed by RT-PCR with primers specific to mouse Cdh3 (Fig. 1b). We refer to the cells as ‘E-cells’ and ‘P-cells’ for cells with tetracycline inducible co-expression of GFP and E-cadherin, or mCherry and P-cadherin, respectively. When cultured separately and without tetracycline to induce cadherin expression, E- and P-cells showed only moderate cell-cell adhesion (Fig. 1c), growing as similar, evenly spread-out lawns. With tetracycline, each line formed adhesive clusters, the E-cells showing smoother-edged and better-defined islands, illustrating the difference in strength of homophilic adhesion between the two cadherins (E-E > P-P^27^). As expected for cadherin-mediated adhesion, this change did not take place in Ca^2 + ^-free medium (Fig. 1c). Wild-type cells showed no change in morphology upon tetracycline treatment (Supplementary Fig S1).

**Figure 2:**
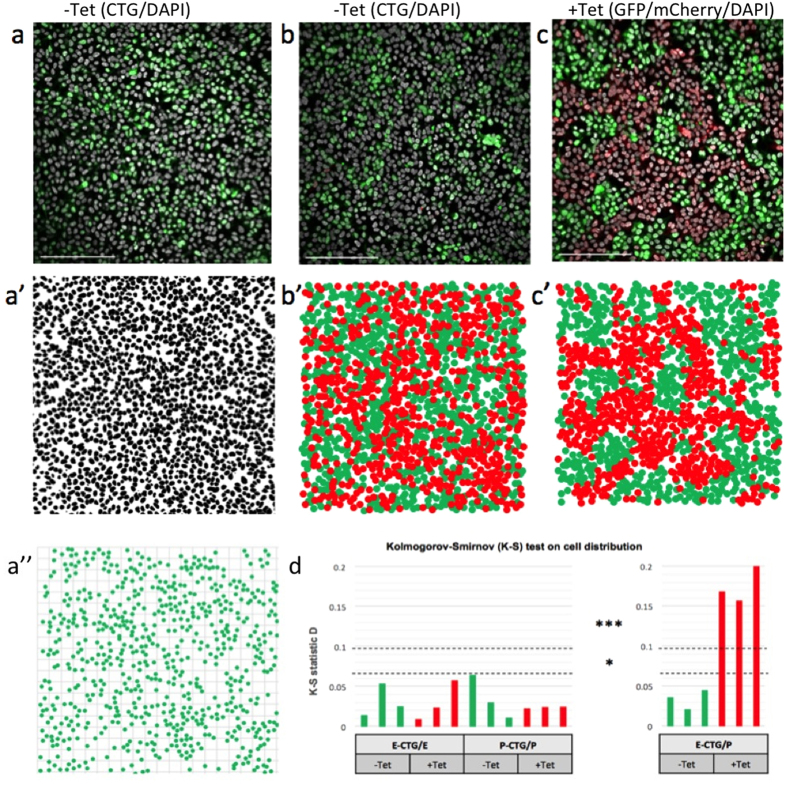
Analysis of cell distributions. (**a**) To verify that patterns did not form in homomixes of cells, an equal mix of E-cells and CellTracker Green (CTG)-labelled E-cells was made (**a**), and the positions of all nuclei (DAPI-stain: a’) and green cells’ nuclei (**a”**) were identified by image analysis and then used for statistical analysis. Similar images were made for P-cells (not shown). (**b**) To verify that patterning would not occur without tetracycline-mediated induction of adhesive differences, E-cells were treated with CellTracker Green (CTG) before mixing with P-cells at a 1:1 ratio. This labelling was necessary because, without tetracycline, there would be no expression of fluorescent marker proteins. After 24 h of culture with or without tetracycline, cells were fixed and stained with DAPI and cell coordinates were mapped in ImageJ (**b’**) for statistical analysis. (**c**) With tetracycline induction, a similar mix of cells revealed E-cells by GFP (and CTG) and P-cells by mCherry: again, nuclear coordinates were mapped for analysis (**c’**). (d) Kolmogorov-Smirnoff statistical analysis of distributions showed no evidence for patterning in homomixes (E-CTG with unlabelled E, or P-CTG with unlabelled P), or in heteromixes (E-CTG/P) without tetracycline, but strong evidence for patterning (non-random distribution) in heteromixes (E-CTG/P) when induced with tetracycline.

Before testing the ability of the mixed cell populations to generate differential-adhesion patterns, we verified that induced cells of a single type would not rearrange themselves into visible patterns simply through homo-cadherin interactions. Populations of the same cell type were split equally, marked with CellTracker probes and re-combined (Fig. 2a), with the position of all cells (Fig. 2a’) and just green cells (Fig. 2a”) being recorded for analysis. After seeding and induction with tetracycline for 48 h, no patterns were generated in these homotypic mixes (Supplementary Fig. 2). This also demonstrated that the cell marking technology did not itself induce patterning.

### Two-dimensional patterning

To determine whether engineered cells would form patterns when mixed and induced, we cultured E- and P-cells in equal ratio in the absence (Fig. 2b,b’) or presence (Fig. 2c,c’) of tetracycline. To be able to differentiate cells when uninduced, we used CellTracker Green to mark E-cells. Quadrat analysis was used to compare each cell distribution to a random Poisson distribution, to test if the marked population exhibited a patterned (non-random) distribution for each condition. Application of tetracycline for 24 h induced clear visual patterning (Fig. 2c), which was absent in non-induced controls (Fig. 2b). For statistical analysis we used a Kolmogorov-Smirnov (K-S) test, the null hypothesis of random distribution being rejected if the resulting K-S statistic D was > 0.0975, which corresponds to a 99.9% confidence threshold. The distribution of cells after 24 h in culture of the uninduced control cells was not statistically different from a random spatial distribution (for three experiments, D_1_ = 0.0357, D_2_ = 0.0208, D_3_ = 0.0446: Fig. 2d) whereas the distribution of the induced cells was non-random (D_1’_ = 0.1683, D_2’_ = 0.1566, D_3’_ = 0.1998; all exceeding the 0.0975 threshold: Fig. 2d). In other words, the induced cells exhibited a pattern.

**Figure 3:**
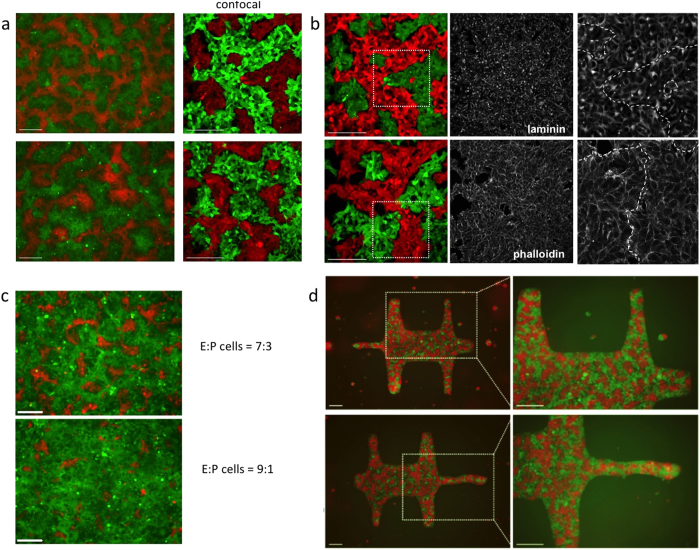
2D cellular patterning through differential adhesion. (**a**) Cellular patterns formed after 48 h of tetracycline-induced cultures and captured with standard epifluorescence (left column) and confocal (second column) microscopes. The two set of images, from separate runs of the experiment, show how the general character of the pattern is reproducible but not the precise detail of the pattern (as the general character of human fingerprints is a feature of all people, but the precise fingerprints of individuals are unique). (**b**) Laminin- and phalloidin-stained patterns showed no particular organization at phase boundaries: the monochrome images on the right of each row show the boxed area in the colour image at higher magnification, and the shape of the boundary has been marked (by inspection of the colour image). This boundary cannot be discerned in the middle image of each row, making the point that there is no obvious cellular reorganization there. (**c**) Varying starting cellular mix ratios (7:3 and 9:1) yielded different patterns in tetracycline-induced cultures. (**d**) Bounded patterns formed on micro-fabricated adherent shapes showed no particular re-organization along field boundaries. Unless indicated, fluorescence represented in green and red in these images originated from GFP and mCherry respectively. Scale bars: 200 μm.

After 48 h, equal ratio mixes showed extensive cellular patterning (Fig. 3a). E-cells (GFP) and P-cells (mCherry) were clearly segregated and formed elongated islands or stripes. Seeding cells close to confluence enabled us to minimize the effect of cellular growth on pattern formation by limiting localized clonal growth that could be mistaken for patterned structures. The pattern could be detected using the cell markers (Cell Tracker and the fluorescent proteins induced by tetracycline) but not by examination of cells either by phase contrast, anti-laminin or phalloidin staining (Fig. 3b), suggesting that the patterned cell sheet retained its physical integrity. When one cell strongly outnumbers the other, spots formed instead of stripes (Fig. 3c).

The boundaries of multicellular growth might convey specific properties to cells against the boundary^23^,^28^. To provide a visible boundary rather then the difficult-to-image edge of a culture well, we used printed shapes fabricated on adhesive shapes micro-patterned on glass slides, the shapes being suitable for cell attachment and their surroundings not. The shapes were the approximate shape of an animal pelt, about 2 mm across. Stripe patterns formed as before (Fig. 3d). Both cell types were capable of occupying a position at the boundary and the proportion of boundary occupied by each cell type was proportional to the final ratio of the populations across the whole shape, within an error margin of about 5%. There were therefore no apparent edge effects.

### Three-dimensional patterning

**Figure 4:**
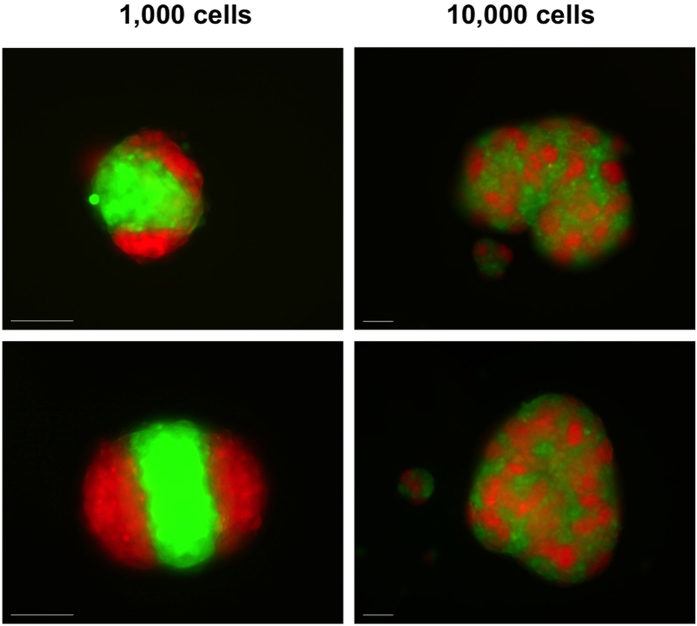
3D patterns. 3D patterns formed in cell aggregates of different sizes: 1,000 or 10,000 cells were suspended in hanging drops and cultured for up to 5 days. Small aggregates showed complete cell sorting, while larger aggregates produced patterns similar to those observed in 2D. Again, the images indicate the reproducibility of the general character of the pattern, and the variability of the precise details. Scale bars: 200 μm.

Having characterized the adhesion-induced patterns in 2D, we explored the formation of these patterns in 3D. To do so, we cultured equal cell mixes of different total cell numbers in hanging drops for up to 5 days. With small cell aggregates (around 1,000 cells), the two cell populations were able to sort almost entirely and formed well segregated clusters, as reported in previous publications^27^,^29^. E-cells formed the core of the aggregates, with P-cells rearranging to their periphery. This reflects previous observations that E-cadherin homotypic adhesions are stronger than P-cadherin homotypic adhesions^27^. In larger aggregates (around 10,000 cells), complete segregation could not be reached. As a result, intricate 3D stripe patterns were formed, similar to their 2D counterparts (Fig. 4).

## Discussion

Taking as its foundations reports that have shown that cadherin-mediated adhesion can drive cell sorting^24^,^25^, this study has built a system that generates rich and complex patterns by phase separation. The system is able to drive mammalian cells to generate intricate 2- or 3-dimensional patterns *de novo*, with no need for the experimenter to provide pre-existing positional information. It therefore provides an alternative, engineered method of pattern generation that is separate from, but that can be compared with, natural evolved mechanisms such as reaction-diffusion^6^,^7^,^30^. In both cases, there are limits of scale (in reaction-diffusion, a field too small compared with the diffusion constants of the molecules results in only one zone of activator; in phase separation an aggregate of cells too small results in complete separation and only one zone of each cell type). Also, in both the engineered and natural systems, while the general character of the pattern (spots / stripes) can be deduced from starting conditions, the precise pattern is not predictable in detail and not reproducible (each run of our experiments produced a different detailed pattern; the natural fingerprints of even monozygotic twins are easily distinguished^31^). As well as allowing researchers to explore an alternative to common, evolved systems for *de novo* pattern generation in animal cells, the construction of a synthetic, inducible patterning system such as this is a step towards the engineering of self-organizing, multicellular synthetic tissues.

## Materials and Methods

### Methods

#### Constructs

Mouse Cdh3 was amplified from plasmid pβact-Pcad [BCCM/LMBP #2766]^27^. The mCherry gene was cloned from pCherryPicker2 (Clontech). Cdh3 and mCherry were linked through a 2A peptide sequence by fusion PCR before insertion in pDONR-221 kindly donated by Agnès Roure^32^ and shuttled into pT-REx-DEST30 (Invitrogen) through Gateway^®^ recombination according to manufacturer’s instructions, to create plasmid pTREx-mCherry-2A-Cdh3.

#### Cell Culture, Transfections and Clonal Selection

T-REx-293 cells (Invitrogen) were maintained in T-REx-293 culture medium, which consisted of DMEM (Gibco 41966) supplemented with 10% FBS (Biosera) and 5 μg/mL blasticidin (Gibco), at 37 °C and 5% CO_2_. Cells were transfected in 24-well plates using 1 μg plasmid and 2 μL lipofectamine 2000 (Invitrogen), in 100 μL Opti-MEM (Gibco) for each well. Cells were then selected using 800 μg/mL G418 (Sigma) for 2 weeks. Stably transfected cells were maintained and tested in T-REx-293 culture medium with 200 μg/mL G418. After transfection of T-REx-293 cells with pTREx-mCherry-2A-Cdh3, individual clones were isolated and tested under induction with 1 μg/mL tetracycline (Sigma). Clone THAD3-34 was selected according to its fluorescence intensity and homogeneity, and is referred to as ‘P-cells’ in the main text of this report. We have described the similar production of clone THAD1-34, referred to as ‘E-cells’ in the main text, in a previous paper^9^.

#### RT-PCR

mRNA was extracted from wild-type T-REx-293 cells and P-cells, induced or uninduced with 1 μg/mL tetracycline for 48 h, following RNeasy kit’s instructions (QIAGEN). RT-PCR was performed on 2 μg of RNA with MLV-RT (Promega). cDNAs from Cdh3 and β-actin as internal control were amplified with specific primers over 20 PCR cycles. Primers for mouse Cdh3: Cdh3-1234F (CTCCCGACAGCCACTGCCAC) and Cdh3-1583R (AGCAGGAGGGTCCCAGTGC); and for β-actin: β-actin_F (CTGGGACGACATGGAGAARA) and β-actin_R (AAGGAAGGCTGGAARAGWGC).

#### 2-D pattern formation

E-cells and P-cells were seeded at indicated ratios on 6-well plates (or on glass coverslips for immunostaining) and 1 μg/mL tetracycline (Invitrogen) was added to the culture medium for 24 h or 48 h to induce patterning. (1 μg/ml is about 10x the concentration needed to induce the constructs, and was chosen so that enough tetracycline would be left even after several half-lives, approx. 24 h according to the manufacturer). When specified, cells where treated before seeding with CellTracker CMFDA (Green, CTG) or CellTracker CM-DiI (Red, CTR, Life Technologies) following manufacturer’s instructions. For calcium-free conditions, DMEM no-calcium medium (Gibco) was used.

#### 3-D pattern formation

E-cells and P-cells were mixed in equal proportions. Cell concentration was adjusted to 10^5^ or 10^6^ cells/mL in culture medium supplemented with 1 μg/mL tetracycline. 10 μL drops were deposited on the inverted lid of a 96-well plate and the corresponding wells were filled with culture medium to serve as hydration chambers. Cell aggregates were monitored and imaged on the lid with an inverted fluorescence microscope.

#### Cell Imaging and immunostaining

Live cell images were acquired using a Zeiss AxioObserver D1 inverted fluorescence microscope with AxioCam MRm and 10x or 20x objectives. Filter excitation (Ex) and emission (Em) bandpass specifications were as follows (in nm): GFP/CellTracker CMFDA (Ex: 470/40, Em: 525/50); mCherry/CellTracker CM-Dil (Ex: 545/25, Em: 605/70). For immunostaining, cells were cultured on sterile coverslips and fixed with 4% paraformaldehyde. After permeabilization with 0.1% Triton X-100, cells were incubated with different stains and mounted on glass slides with glycerol: 300 nM DAPI (4’,6-Diamidino-2-Phenylindole); 0.1 μg/mL phalloidin, coumarin labeled (Sigma); 5 μg/mL anti-laminin (Sigma) coupled with Alexa Fluor^®^ 647 (Abcam). Confocal images were acquired using a Nikon A1R confocal system combined with Ti:E inverted microscope with 20x objective. Filters bandpass specifications were as follows (in nm): DAPI (Ex: 401, Em: 445/50); FITC (Ex: 488, Em: 525/50); TRITC (Ex: 561, Em: 595/50). Where necessary, fluorescence contrast was enhanced (across the whole image) using the GIMP picture editor. This made no difference to the features/ patterns illustrated, to which absolute levels of fluorescence are not relevant, but simply made the red and green colours richer. All scale bars are 200 μm across.

#### Statistical analysis

Image analysis was performed in imageJ (mapping of nuclei coordinates, identification of green-fluorescing cells, area measurements). Quadrat analysis was performed: image fields were divided in 400 quadrats and the number of cells per quadrat was recorded. The quadrats were grouped according to the number of cells they contained and the frequency for each quadrat group was calculated. The statistical Kolmogorov-Smirnov test was performed in order to compare cell frequency distributions to a random Poisson frequency distribution by measuring the absolute, largest difference between cumulative frequencies: the D statistic value. The null-hypothesis was rejected and the cell distribution considered patterned if the D statistic value was above the 99.9% confidence threshold (0.0975). The cell distribution was considered random if the D statistic value was below the 95% confidence threshold (0.068), indicating the distribution was not significantly different from a Poisson distribution.

#### Micropattern fabrication

Glass slides were amino-salinized for 1 h in ethanol after piranha acid treatment. S1805 photoresist (Shipley) was spin-coated on the aminized slides and an animal-pelt-shaped mask was placed over the slides before exposing the photoresist to UV light for 4 s. The slides were developed for 40 s, oxygen plasma-treated for 3 min and sonicated in water for 2 min. Polyethylene glycol (PEG)-silanization in dry toluene was performed for 2 h to prevent cells to adhere outside of the photoresist-treated areas. The slides were then dried in dimethylsulfoxide (DMSO) and isopropyl alcohol to remove residual photoresist. Dried slides were stored in vacuum.

## Additional Information

**How to cite this article**: Cachat, E. *et al.* 2- and 3-dimensional synthetic large-scale *de novo* patterning by mammalian cells through phase separation. *Sci. Rep.* **6**, 20664; doi: 10.1038/srep20664 (2016).

## Supplementary Material

Supplementary Information
