# Supplementary material for: 2- and 3-dimensional synthetic large-scale de novo patterning by mammalian cells through phase separation
Source: Sci Rep. 2016 Feb 9;6:20664. doi: 10.1038/srep20664 (PMC4746622; doi:10.1038/srep20664)
Supplement: Supplementary Information [file srep20664-s1.pdf]

2- and 3-dimensional synthetic large-scale de novo patterning by mammalian cells through phase separation.

Elise Cachat, Weijia Liu, Kim C. Martin, Xiaofei Yuan, Huabing Yin, Peter Hohenstein & Jamie A. Davies.

SUPPLEMENTARY FIGURES

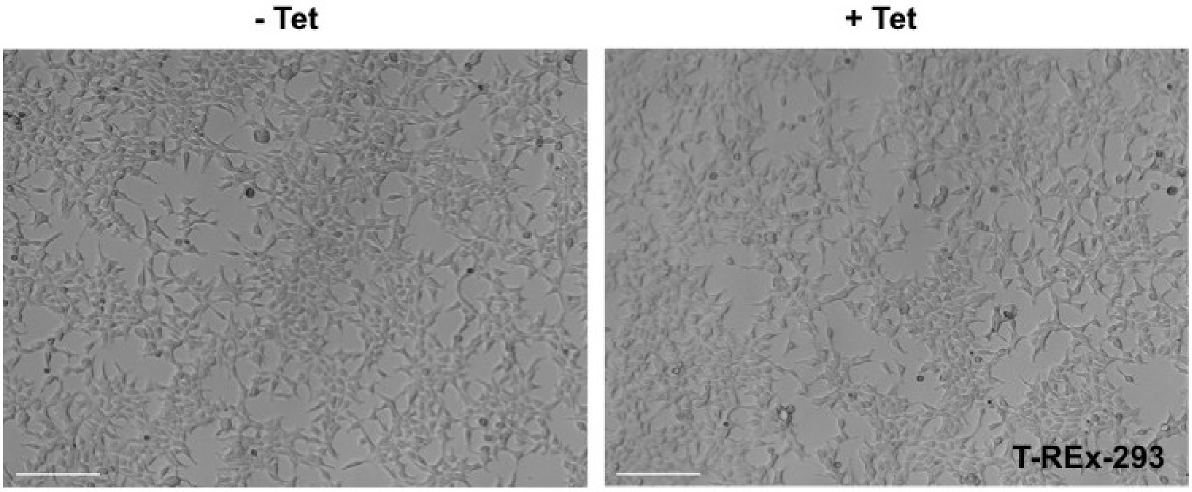

Supplementary Figure 1

Wild-type T-Rex-293 cells with or without tetracycline induction. T-Rex-293 cells did not show morphological changes in response to 48 h culture in the presence of 1 µg/mL tetracycline. Scale bars: 200 µm.

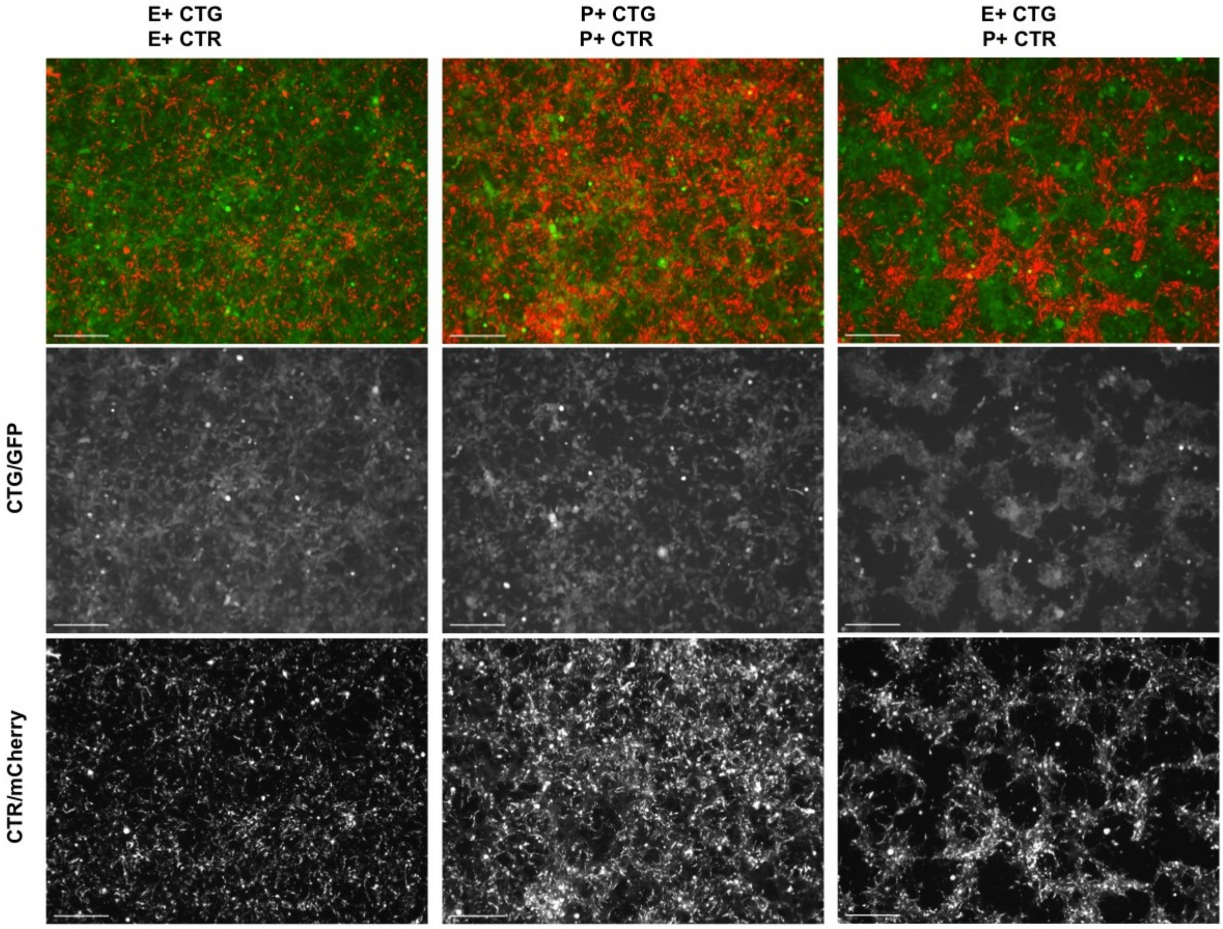

Supplementary Figure 2

Homomixes do not form patterns after tetracycline induction.

For each cadherin-expressing clone, cells were split in two subpopulations and marked with CellTracker CMFDA (Green, CTG) and CellTracker CM-Dil (red, CTR). Homomix cultures were set up after combining subpopulations expressing the same cadherin but marked with different colour dyes (i.e. E+CTG/E+CTR and P+CTG/P+CTR). After 48 h of culture under tetracycline induction, no patterns were observed in these homomixes in comparison to a heteromix (E+CTG/P+CTR) cultured under the same conditions.
